# Peritoneal Dialysis in Austere Environments: An Emergent Approach to Renal Failure Management

**DOI:** 10.5811/westjem.2018.3.36762

**Published:** 2018-04-05

**Authors:** Chad Gorbatkin, John Bass, Fredric O. Finkelstein, Steven M. Gorbatkin

**Affiliations:** * Madigan Army Medical Center, Department of Emergency Medicine, Tacoma, Washington; † Yale University, Department of Nephrology, New Haven, Connecticut; ‡ Atlanta VA Medical Center, Emory University, Department of Nephrology, Atlanta, Georgia

## Abstract

Peritoneal dialysis (PD) is a means of renal replacement therapy (RRT) that can be performed in remote settings with limited resources, including regions that lack electrical power. PD is a mainstay of end-stage renal disease (ESRD) therapy worldwide, and the ease of initiation and maintenance has enabled it to flourish in both resource-limited and resource-abundant settings. In natural disaster scenarios, military conflicts, and other austere areas, PD may be the only available life-saving measure for acute kidney injury (AKI) or ESRD. PD in austere environments is not without challenges, including catheter placement, availability of dialysate, and medical complications related to the procedure itself. However, when hemodialysis is unavailable, PD can be performed using generally available medical supplies including sterile tubing and intravenous fluids. Amidst the ever-increasing global burden of ESRD and AKI, the ability to perform PD is essential for many medical facilities.

## INTRODUCTION

Peritoneal therapies historically focused on the removal of accumulated fluids.^1^ In 1923 Dr. Georg Ganter, emboldened by animal studies, performed peritoneal dialysis (PD) on an anuric patient, which temporarily improved the patient’s mentation.^2^ This proof of concept spurred decades of further research, culminating in the successful treatment of acute renal failure via peritoneal lavage by Seligman, Frank and Fine in 1946.^1^

Subsequently, the production of malleable dialysis tubing and standardized dialysate improved patient outcomes. Mortality rates for acute renal failure treated by PD dropped below 50%, and acceptable treatment durations grew from days to months.^1^ These advancements were applied to casualties of the Korean War and Vietnam War, who had significantly better recovery from acute kidney injury (AKI) compared to their World War II counterparts.^3^ The goal of eliminating repeated abdominal wall punctures and continually improving patient outcomes culminated in the Tenckhoff catheter, which was introduced in 1968.^1^ This tunneled device used the latest in materials, reduced complications, and allowed safe PD therapy for extended periods, creating the foundation for modern-day therapy.^1^

Currently used by an estimated 196,000 patients worldwide, PD is heralded for its ease of initiation, conservation of resources, and efficacy.^4^ Accordingly, PD may be a reasonable alternative to hemodialysis (HD) for AKI even when both are available.^5^,^6^ During disaster responses and in resource-limited settings, including Turkey in 1999 and Haiti in 2010 following devastating earthquakes, improvised PD has been performed successfully using general medical resources.^7^,^8^,^9^,^10^ Likewise, without adequate supplies or equipment to sustain HD in the aftermath of Hurricane Katrina in 2005 and in India in 2010, PD was rapidly and safely initiated by trained professionals, including emergency physicians (EP), to manage renal disease.^9^,^11^,^12^ These successes have been re-demonstrated by international programs, including those in Brazil and India, and the Saving Young Lives Program in sub-Saharan Africa and Southeast Asia, which provide vital PD care in low-resource settings.^5^,^13^,^14^,^15^ Similarly, PD has been vital to the care of chronic and acute renal injury patients alike during contemporary military operations in Iraq and Afghanistan, as well as during the 2014 Syrian humanitarian crisis.^7^,^8^,^16^ From emergent initiation following natural disasters to routine use in non-austere settings, PD has become a keystone in managing renal insufficiency worldwide; its use is aided by the International Society for Peritoneal Dialysis (ISPD) Guidelines, which promote safe and effective therapy.^17^

## INDICATIONS FOR PERITONEAL DIALYSIS IN AUSTERE SETTINGS

### Acute Kidney Injury

General indications for dialysis are the same in austere and non-austere settings. An example renal replacement therapy (RRT) protocol for AKI management highlights the impact of electrolyte and metabolic data, if available, on deciding to initiate therapy (Table 1). Common indications for PD in austere settings include severe acidosis, hyperkalemia, and uremia.

Rhabdomyolysis with myoglobinuric AKI is a common indication for urgent PD therapy, particularly following crush injuries that occur, for example, during earthquakes.^18^ The initial treatment includes correcting electrolyte abnormalities and maintaining renal tubular flow with volume resuscitation, with a goal urine output of 3 mL/kg/hr.^19^ Delays in care can result in anuric AKI with life-threatening acidosis, multi-organ failure and hyperkalemia, and may prompt emergent PD prior to transport for HD.

Shock is an important cause of acute tubular necrosis and life-threatening AKI.^7^,^8^,^20^ Despite aggressive resuscitation, these patients are at high risk of progressive AKI and subsequent severe acidosis and hyperkalemia. Evacuation may not be feasible prior to the development of life-threatening indications for dialysis, necessitating immediate management. For neonatal and pediatric AKI, including from diarrheal illness and sepsis, PD is the preferred therapy.^12^

Hypervolemia and toxin clearance in isolation may also require urgent-start PD. Using high dextrose dialysate, volume can be removed. There is significant variability in toxin clearance via dialysis, with large or extensively plasma protein-bound molecules more difficult to clear. However, PD has been used alone or as a bridge to HD for potentially lethal exposures amenable to dialysis treatment, though PD would be expected to be less effective than conventional HD.^21^,^22^

### During War or Natural Disaster

Patients requiring PD in austere settings include those previously undergoing chronic PD or HD therapy, and those newly meeting dialysis criteria. Consideration of timeline to HD access and of clinical data, including severity of illness, patient volume status, and electrolyte profile, may dictate the immediate or eventual initiation of PD.

### Contraindications

Relative contraindications to PD initiation include recent abdominal surgery, diaphragmatic injury, overlying soft tissue infection, and known peritoneal adhesions.^23^ Additionally, patients with severe respiratory failure may not tolerate intraperitoneal fluid.

## ESTABLISHING ACCESS FOR PERITONEAL DIALYSIS

### Catheter Options

**Figure 1:**
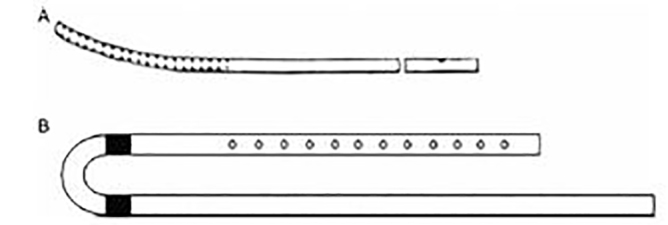
Dedicated peritoneal dialysis catheters are commercially available in rigid (A) or flexible (B) configurations, and typically measure 9.5 French diameter and approximately 37 centimeters in length (Reprinted from Abraham, G et al, A review of acute and chronic peritoneal dialysis in developing countries, Clinical Kidney journal, 2015, Volume 8, Issue 3, Pages 310–317, by permission of Oxford University Press).^15^

Two primary types of PD catheters are commonly used: rigid and flexible. Flexible catheters are preferred when available.^15^ If PD is anticipated, dedicated catheters may be ordered and made available (Figure 1). However, improvised catheters may be the mainstay of PD therapy in austere settings. The Tenckhoff continues to be the gold standard in flexible catheters, based on its higher dialysate flow rates, and fenestrations that make it less prone to obstruction.^25^ Available in single- and double-cuffed designs, the latter is preferred for its additional anchor point in the preperitoneal space, added barrier to infection, and improved overall patient satisfaction.^4^,^7^,^26^ Though more expensive than rigid catheters, and requiring a tunneled insertion, the flexible catheter is associated with lower rates of complication.^7^

Rigid catheters are inserted using a sharp, removable trocar in a non-tunneled fashion, which allows for quicker placement.^25^ However, they are also prone to higher rates of complication, including dialysate leakage, and increased occurrence of bowel or bladder perforation upon insertion.^25^ While a feasible option, especially for short-term management, the flexible Tenckhoff catheter is preferred. In austere settings, dedicated PD catheters will often be unavailable, and any sterilized medical tubing can be used. Alternative materials, such as nasogastric tubes, suprapubic catheters, pediatric chest tubes and central venous catheters have been effective for initiating PD in resource-limited environments.^27^ Clinical data including anticipated duration of therapy, availability of supplies, and patient body habitus may dictate catheter selection.

### Catheter Placement

The most experienced provider should place the catheter using best available resources, and if available, consultation should always be sought. General surgeons, interventional radiologists, and nephrologists commonly place PD catheters, but EPs and other procedurally experienced physicians can place PD catheters in austere and non-austere settings alike.^3^,^8^ Percutaneous catheter insertion is standard in austere settings, with catheters placed blindly or under ultrasound or radiographic guidance. Percutaneous placement does not require specialized surgical equipment or general anesthesia.^28^ General sterile technique and analgesia are necessary, with moderate sedation also encouraged. Pre-procedural intravenous (IV) antibiotics, such as vancomycin are recommended to decrease the risk of peritonitis.^15^,^29^

Catheter type dictates the optimal approach. If an improvised flexible catheter is being used, the placement requires a midline incision 2 cm below the umbilicus, blunt dissection to the linea alba, puncture through the linea alba with a rigid catheter, infusion of a small volume of dialysate, insertion of a guidewire through the initial catheter, and dilation using Seldinger technique to the final catheter.^28^ For rigid catheters, placement includes anesthetizing the point of insertion immediately lateral to the umbilicus, and advancing the device with the aid of a pointed trocar, directed caudal toward the iliac fossa.^17^

**Figure 2:**
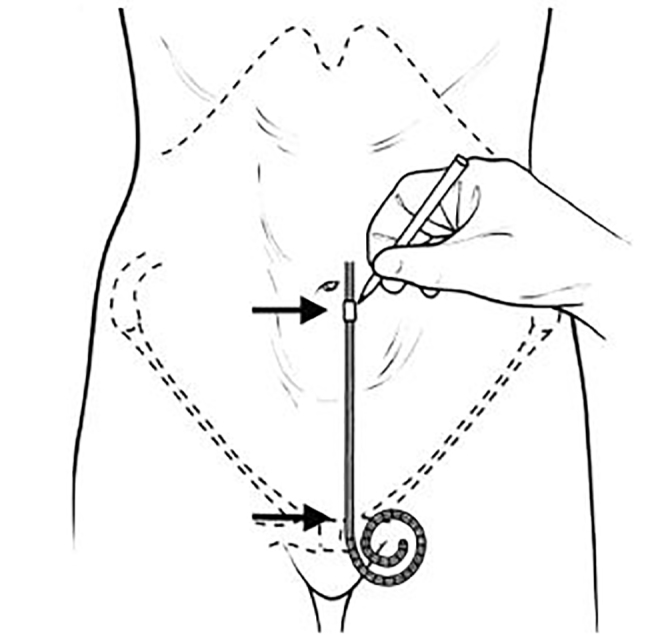
In the supine patient, prior to surgical placement, the upper border of the distal catheter coil should be aligned with the superior border of the pubic symphysis, and the corresponding cuff insertion sites marked (with a handheld marker as shown). This technique helps limit catheter tip migration by positioning the device at the inlet of the true pelvis (Reprinted from Kidney International, Volume 70, Crabtree, JH, Selected best demonstrated practices in peritoneal dialysis access, Pages S27–S37, 2006 with permission from Elsevier).^28^,^30^

When using a dedicated PD catheter, with the patient in a supine position, the upper border of the distal catheter coil should be aligned with the superior border of the pubic symphysis (Figure 2).^28^,^30^ This position correlates with the boundary of the true pelvis, and helps limit catheter tip migration. The catheter should be oriented cephalad, approximately 3 cm lateral of midline, and the deep- and superficial-cuff points should be marked on the anterior abdominal wall.^28^ A small skin incision is made at the deep-cuff point, and blunt dissection is completed down to the abdominal rectus sheath. Using Seldinger technique, a guidewire followed by dilator and peel-away sheath are advanced into the peritoneal cavity. The catheter is then advanced through the sheath, which is gradually peeled away. The catheter’s free end is then tunneled via blunt dissection to the superficial-cuff point, where it exits the subcutaneous tissue and is available for use, following closure of the skin incisions.^28^

When available, this technique may be assisted by fluoroscopic or ultrasound guidance for real-time, intraoperative monitoring.^31^ In a small, prospective study of ultrasound-guided percutaneous catheter placement, providers demonstrated comparable success rates to the surgical technique, without any immediate procedure-related complications.^31^,^32^ Continued investigation into alternative techniques for PD catheter placement has improved outcomes, while limiting the use of certain costly, prohibitive materials.^33^

Open or laparoscopic surgical placement allows direct visualization of catheter tip placement, and enables adhesiolysis and omentopexy to reduce the likelihood of catheter tip obstruction or impaction.^28^ Following insertion of the catheter at the previously marked, deep-cuff point, a subcutaneous tunnel is created toward the superficial-cuff point, the catheter is secured, and operative sites closed. While no studies have demonstrated a significant difference between surgical and percutaneous placement with respect to complications and survival at one year, each modality should be vetted against available resources and personnel.^34^

### Patients with Indwelling PD Catheters

PD catheter connectors are not universal. In patients with established catheters, use of their indwelling device may require a specific adaptor. If an adaptor is unavailable and an improvised adaptor cannot be constructed, the decision must be made to either modify (and possibly compromise) the existing catheter or to place a second, improvised catheter.

## DIALYSATE

Dialysate is a solution of water, electrolytes and osmotic agents, formulated to aid in the clearance of metabolic waste while stabilizing acid-base or electrolyte derangements.^35^ Commercially available solutions, such as Physioneal®, Dianeal® and Nutrineal® by Baxter, are prepared under stringent aseptic standards, but might not be universally available.^36^ Accordingly, dialysate may be prepared from IV fluids and tailored to the clinical indication (see Tables[Table t1-wjem-19-548][Table t2-wjem-19-548][Table t3-wjem-19-548]).^5^,^37^ Peritoneal dialysate typically contains sodium (131–134 mmol/L), chloride (95–105 mmol/L), bicarbonate plus lactate (35–41 mmol/L), dextrose (1.5, 2.5, or 4.25%), and zero potassium. Dialysate can be mixed using normal saline with additives including sodium bicarbonate and dextrose with water, but produces a notably sodium-rich solution.^38^

Lactated Ringer’s solution (LR) has a similar electrolyte profile to commercial dialysate but contains 4 mEq/L of potassium. Accordingly, the addition of an osmotic agent, such as 50 mL of 50% dextrose (D50) per liter LR, will yield a potassium-containing dialysate solution ready for use.^3^ For the hypervolemic patient, volume removal may be further augmented by adjusting dialysate osmolality via the addition of dextrose. For example, dextrose concentrations increase 1% by adding 20mL of D50 per liter, targeting the 1.5–4.25% dextrose concentration found in most commercial dialysates.^38^ Through frequent electrolyte monitoring of the effluent and serum, the dialysate can be adjusted, e.g., by adding potassium to dialysate at serum potassium concentrations less than 4 mmol/L.^34^ Of note, when prepared from individual components, special considerations should be made to ensure sterile technique. With each addition to the dialysate prescription, the risk of iatrogenic infection increases, which represents a modifiable risk to patient safety and outcomes.^39^,^40^ Additionally, antibiotics including aminoglycosides, cephalosporins and vancomycin can be added to each PD exchange for prophylaxis or treatment. To prevent PD catheter obstruction, heparin can be added to each liter of dialysate, with a typical dose of 500 units per liter.^38^

## DIALYSIS PROCESS

### Urgent-Start Peritoneal Dialysis

Following placement of the indwelling catheter, PD may be accomplished by several means. PD is either an automated or non-automated process. The abdominal cavity is filled with a prescribed volume of dialysate; the solution is allowed to dwell for a period of time, during which the peritoneum functions as an exchange barrier for fluids and solutes before the dialysate is drained. For non-automated PD, the most common technique includes attaching a three-way stopcock to the improvised catheter, infusing 1–2 L of dialysate in an adult, with dwell times of 2–4 hours, four times per day.^39^ In pediatric cases, 10–20 mL/kg of dialysate is appropriate, with total exchange times of 60–90 minutes, incorporating 30–40 minutes of dwell time.^17^,^41^,^42^ Drainage may be performed by gravity or aspiration of the dialysate. Case reports from military providers in Iraq and Afghanistan suggest small volume dwells of 500–1,000mL for 2–4 hours are also reasonable, with subsequent optimization based on volume status (e.g. altering dialysate dextrose).^7^,^42^ Dialysate volume usage for adult AKI may range from 4–70 liters per day, depending on modality and targets of therapy.^6^,^8^,^39^ Frequency and duration of therapy can be tailored to clinical circumstances, in consultation with ISPD Guidelines and a nephrologist, if available.^17^

While less frequently used in developed countries, continuous ambulatory PD is the primary method in 59% of the nearly 160,000 PD patients worldwide.^4^ Furthermore, successful initiation of non-automated PD is well-documented in regions with limited medical infrastructure. Military physicians have successfully implemented non-automated PD in austere, deployed settings for critically ill patients, while reports from post-earthquake Haiti and Turkey have highlighted similar benefits in low-resource environments.^7^,^8^,^10^,^18^

Automated peritoneal dialysis (APD) refers to the electricity-dependent mechanical infusion and drainage of dialysate, and has limited utility in austere environments based on its use of large dialysate volumes and the requirement of a dependable energy source.

Throughout PD therapy, electrolytes, especially potassium, should be measured frequently. Daily electrocardiograms are also recommended, and may serve as an alternative for hyperkalemia screening if laboratory testing is unavailable.^17^ The adequacy of PD is best assessed by the absence of hypertension, edema, and electrolyte and acid-base abnormalities.^37^ Other markers of adequacy, such as weekly kT/V of urea where k is the clearance per unit time of urea, T = time and V = volume of distribution of urea, have been adapted for PD prescriptions, targeting kT/V >2.1 in AKI or >1.7 in ESRD.^6^,^34^,^39^

As above, the prescription required to achieve adequate PD is not precisely defined. Patient characteristics, including total surface area and peritoneal transport kinetics, should be considered when adjusting this regimen, though this is seldom known in austere settings. Of note, some patients have intrinsically high rates of diffusive peritoneal transport, and may benefit from shortened dwell times at increased frequency to promote clearance and limit excessive resorption of dialysate.^17^ Thus, sometimes the non-intuitive intervention of shortening dwell times may be required to increase volume removal and dialysis efficacy. PD prescriptions should be optimized based on available resources, subject matter experts, and the ISPD Guidelines.

## COMPLICATIONS

Advancements in PD catheter materials and placement technique ensure a safety profile comparable to other common invasive procedures. Complications from PD are classified as “early” and “late,” and correspond to the first days following placement, or thereafter.^43^ Comprehensive reviews of complications are essential but beyond the scope of this paper.^44^,^45^,^46^,^47^

During catheter placement, significant injury is a rare but important potential complication. Significant hemorrhage is often confined to the skin or subcutaneous tissue; it is mitigated by blunt dissection during catheter placement, and the provider’s procedural experience.^43^ Similarly, bowel or bladder injury, especially during rigid catheter placement, may be limited or rapidly identified by the use of ultrasonography or radiography.^48^ Post-operative vital sign abnormalities or progressive abdominal pain in the awake patient should raise concern for viscus injury.

Intraperitoneal and exit-site infections, while limited by sterile technique, may present with local erythema and discharge at the operative site. While uncomplicated cases may be managed with oral antibiotics, surgical debridement and hardware removal should be considered for complicated cases.^43^

Peritonitis remains the most common late complication in PD, with varying incidences up to 0.24 episodes per patient per year.^45^ While infection risks are minimized by pre-procedural prophylactic antibiotics, often with vancomycin or cephalosporins, signs of peritonitis should be promptly investigated and treated with two weeks or more of antibiotics.^43^,^49^ During antibiotic therapy, prophylactic oral nystatin or fluconazole should be considered to reduce the risk of concomitant fungal peritonitis.^49^ In hemodynamically stable patients, dialysis therapy should be continued while treatment is administered through the peritoneal catheter.^43^ In cases of fungal peritonitis, lack of improvement following five days of antibiotic therapy, or relapsing/refractory peritonitis, removal of the device is strongly recommended.^43^

Dialysate leakage is a frequent complication of this procedure, and is often related to initiating therapy soon after catheter placement, or using a large dialysate volume.^43^ Leakage may be minimized by allowing 10–14 days for tract healing following surgery, which will generally not be feasible for austere urgent-start PD. Of note, certain placement centers have had excellent success with urgent-start PD in the non-ambulatory setting, with leakage rates as low as 2% within the first month.^42^,^50^ This complication is often managed conservatively with reduced dialysis frequency or volume, and rarely with repeated surgical intervention.^51^

Hydrothorax is an uncommon early and late complication of peritoneal catheter placement, though it may cause dyspnea and respiratory insufficiency in PD patients, requiring thoracentesis or thoracostomy.^52^ Small-volume PD exchanges may be helpful to minimize the accumulation of the hydrothorax, though surgical intervention via pleurodesis or thoracotomy with diaphragm repair may be indicated, if available.^44^

Finally, catheter tip migration can occur following placement and result in obstructed dialysate drainage and discomfort. If the catheter is improperly placed or secured, the device’s inherent shape-memory may displace the catheter tip as it reverts back to its native configuration.^51^,^53^ Migration into the omentum increases the risk of local trauma from mechanical irritation or forceful attempts at flushing the catheter.^43^,^54^ Depending on the technique for catheter placement and provider comfort, prophylactic omentectomy or omentopexy may reduce this complication**.**

## OUTCOMES

PD is a life-saving therapy in austere and non-austere settings for both AKI and ESRD, but data regarding long-term outcomes are limited. A review of published literature by Chionh et al. did not identify a significant difference in outcomes between PD and extracorporeal blood purification for AKI.^6^ Patients have regained renal function with long-term survival after urgent-start PD, when used as either primary therapy or bridging therapy to HD or renal transplant. Some patients will improve, with or without renal insufficiency, whereas others will require lifetime RRT or die.^7^ The underlying cause often dictates prognosis of acute renal failure patients, but optimizing outcomes may require early dialysis in the austere setting. A small, prospective study demonstrated no increased incidence of early complications with immediate initiation of PD, and therapy should not be delayed for the acutely ill.^43^ If transport to higher level of care of HD is unavailable, PD using available resources may be required.

Incident, or abrupt-start, PD patients have a nearly 87% one-year survival rate overall.^55^ Furthermore, there is no significant difference in mortality between continuous ambulatory PD (CAPD) and APD for incident patients immediately following initiation, and the 11.3% survival rate at 10 years for CAPD patients is likely confounded by underlying patient co-morbidities.^48^,^56^,^57^ A recent trial comparing PD with HD in the management of severe acute tubular necrosis showed comparable metabolic control, mortality rates and renal recovery rates, and supports PD as an effective, alternative form of RRT.^58^ While the high volumes of dialysate and automated cyclers would likely be prohibitive in austere settings, PD has been proven beneficial in the critically ill.^14^ Finally, the safety and efficacy of PD has successfully expanded its use to austere and resource-limited regions by certain international organizations combatting AKI and ESRD. Such groups, including the Saving Young Lives Project, bring essential supplies, training and support to medical teams serving sub-Saharan Africa and Southeast Asia.^13^,^59^ Through infrastructure development, the survivability and total recovery from AKI in these regions has improved, while also empowering the local medical community to continue effective, safe dialysis practices.

Regardless of the etiology for acute or chronic renal insufficiency, preservation of residual renal function is a fundamental goal of RRT. Patients with continued renal function demonstrate a significant reduction in the relative risk of death, proportional to their glomerular filtration rate (GFR).^56^ While further investigation is warranted, several observational studies have shown a more rapid decline in native GFR with APD compared to continuous non-automated PD.^60^ In case reports detailing the use of non-automated PD for acute renal failure in patients without prior kidney disease, several patients had no long-term clinically significant impairment on renal function, compared to 23% renal recovery rate with high-dose automated PD.^7^,^55^ By helping preserve renal function, PD may limit the severity of acute renal insufficiency and improve outcomes in select patients.

Peritonitis continues to be a major contributor to PD failure, with annual incidence as high as 1.66 per patient at some programs.^40^ This is significantly influenced by duration of therapy, underlying patient comorbidities, and psychosocial demographics.^60^ Continued development of standardized practices, in conjunction with ISPD Guidelines, may minimize the incidence of treatment failure. Mortality rates for ESRD patients undergoing PD continue to decline worldwide.^61^ For ESRD patients managed chronically on PD in the U.S., there has been notable progress in the reduction of adjusted mortality rates, despite the aforementioned 10-year survival rate. Improved techniques in catheter placement and management contributed to the decline in overall mortality rate for PD patients by 25% between 2004 and 2014, to fewer deaths per 1,000 patient-years than their matched HD counterparts.^62^,^63^ This progress supports PD as a feasible alternative to HD therapy, and is gradually being replicated internationally.

For example, Sudan initiated a government-sponsored CAPD program for ESRD patients in 2005, and in a mere 20-month span established a treatment network capable of meeting accepted standards for efficacy and peritonitis rates.^64^ Such programs demonstrate the feasibility of CAPD in resource-limited regions, and will hopefully promote continued international use of this treatment modality. As a means to manage ESRD or treat acute renal failure in austere settings, PD is an option with demonstrated efficacy in diverse settings throughout the world.

## CONCLUSION

Peritoneal dialysis is a time-tested therapy for managing acute renal failure and ESRD. Despite significant improvements in alternative renal replacement therapies, PD remains an affordable, easy-to-initiate, and effective treatment option for patients with impaired kidney function.^67^ For physicians practicing in austere or low-resource settings, PD should be considered a mainstay of renal failure therapy. In consultation with local and international organizations, subject matter experts, and ISPD Guidelines, PD is an essential and life-saving therapy.

Future research should focus on optimizing the safety and efficacy of improvised PD using readily available medical equipment. Additionally, further investigation could be directed toward establishing improvised hemodialysis. To better understand and use PD in austere settings, outcomes analysis from such applications must continue whenever possible.

## Figures and Tables

**Table 1 t1-wjem-19-548:** Indications for emergent renal replacement therapy.^24^

Disturbance	More urgent	Less urgent	Non-urgent
Acid-base	Metabolic acidosis; pH < 7.2	pH 7.2–7.3	pH > 7.3
Electrolytes	K > 6.5 or EKG changes	K 6.0 – 6.5	K < 6.0
Ingestion	Toxin		
Overloaded	Massive anasarca hypoxemic respiratory failure: fiO_2_ > 0.7 urine output < 100mL/24hrs	2–3+ Peripheral edema hypoxemia : fiO_2_ 0.5–0.7 urine output 100–500mL/24hrs	< 1 Peripheral edema urine output > 500mL/24hrs
Urea	Uremic symptoms altered mental status	BUN 60–130	BUN < 60

*K*, Potassium; *FiO_2_*, fraction of inspired oxygen; *BUN*, blood urea nitrogen.

Electrolyte derangements, metabolic factors, and patient characteristics must be taken into account when considering the initiation of peritoneal dialysis. The presence of any ‘More Urgent,’ or three or more ‘Less Urgent’ features should prompt consideration of peritoneal dialysis.

**Table 2 t2-wjem-19-548:** Improvised peritoneal dialysis recipe.^13^ Depending on circumstances and available resources, dialysate of varying dextrose concentrations may be emergently prepared to correct patient metabolic and electrolyte derangements.

	1.45% Dextrose	1.45% Dextrose	1.7% Dextrose	2.5% Dextrose
Plasmalyte B (mL)	1000			
Lactated ringers (mL)		1000		
0.45% saline (mL)			1000	
0.9% saline (mL)				1000
3% NaCl (mL)			60	
5% Dextrose water (mL)				1000
50% Dextrose (mL)	30	30	40	
8.4% NaHCO_3_ (mEq)			40	100

*NaCl*, sodium chloride; *NaHCO_3_*, sodium bicarbonate; *mEq*, milliequivalent.

Plasmalyte B: Na+ 130, K+ 4, Ca_2_+ 0, Mg 1.5, Cl− 110, HCO3 – 27, pH 7.4, Osmolarity 273.

**Table 3 t3-wjem-19-548:** Examples of acute peritoneal dialysis in austere environments^8^,^27^,^65^,^66^ Worldwide, there are a variety of indications for initiation of emergent peritoneal dialysis, which may be accomplished with dedicated or improvised dialysate solutions and catheters.

Location	Indication	Dialysate	Dialysis catheter	Outcome
Ghana	Anuria; urosepsis	Improvised	12fr thoracic trocar catheter	Full recovery
Nigeria	AKI; HUS	Improvised	14fr NG tube	Recovery
Tanzania	AKI; malnutrition	Improvised	Suprapubic catheter	Full recovery
Afghanistan/Iraq	Acidosis; hyperkalemia	Improvised	Abdominal drain	Full recovery
Afghanistan/Iraq	Acidosis; hyperkalemia	1.5% dianeal	Abdominal drain	Lost to follow-up
Afghanistan/Iraq	Acidosis; fluid overload	Improvised	Pediatric chest drain	Full recovery
Afghanistan/Iraq	Fluid overload	4.25% dianeal	Abdominal drain	Death

*AKI*, acute kidney injury; *HUS*, hemolytic uremic syndrome; *NG*, nasogastric.
